# An Approach Based on an Increased Bandpass for Enabling the Use of Internal Standards in Single Particle ICP-MS: Application to AuNPs Characterization

**DOI:** 10.3390/nano13121838

**Published:** 2023-06-10

**Authors:** Antonio Bazo, Maite Aramendía, Flávio V. Nakadi, Martín Resano

**Affiliations:** Department of Analytical Chemistry, Aragón Institute of Engineering Research (I3A), University of Zaragoza, Pedro Cerbuna 12, 50009 Zaragoza, Spain; abazo13@gmail.com (A.B.); maiteam@unizar.es (M.A.); fvnakadi@unizar.es (F.V.N.)

**Keywords:** single particle ICP-MS, bandpass, internal standard, matrix effects

## Abstract

This paper proposes a novel approach to implement an internal standard (IS) correction in single particle inductively coupled plasma mass spectrometry (SP ICP-MS), as exemplified for the characterization of Au nanoparticles (NPs) in complex matrices. This approach is based on the use of the mass spectrometer (quadrupole) in bandpass mode, enhancing the sensitivity for the monitoring of AuNPs while also allowing for the detection of PtNPs in the same measurement run, such that they can serve as an internal standard. The performance of the method developed was proved for three different matrices: pure water, a 5 g L^−1^ NaCl water solution, and another water solution containing 2.5% (*m*/*v*) tetramethylammonium hydroxide (TMAH)/0.1% Triton X-100. It was observed that matrix-effects impacted both the sensitivity of the NPs and their transport efficiencies. To circumvent this problem, two methods were used to determine the TE: the particle size method for sizing and the dynamic mass flow method for the determination of the particle number concentration (PNC). This fact, together with the use of the IS, enabled us to attain accurate results in all cases, both for sizing and for the PNC determination. Additionally, the use of the bandpass mode provides additional flexibility for this characterization, as it is possible to easily tune the sensitivity achieved for each NP type to ensure that their distributions are sufficiently resolved.

## 1. Introduction

There is no unrivaled technique for the characterization of nanoparticles (NPs), and several methodologies are conventionally employed, as they offer complementary information about their chemical (e.g., composition and concentration) and physical (e.g., shape, size, and aggregation) properties [1]. In this sense, single particle inductively coupled plasma mass spectrometry (SP ICP-MS) has been gaining popularity, as it provides information, even at very low concentration levels, on the average particle mass with its distribution, the NP elemental composition, the concentration of ionic species in the matrix, and the particle number concentration (PNC). Additionally, the average particle size and its distribution can be characterized for NPs of well-known shapes and densities [2]. All these possibilities have broadened the scope of the technique, which is now applied in many different fields for the analysis of cosmetics [3], biological tissues [4,5], food [6,7], and environmental samples [8,9,10,11,12], just to mention a few. However, there are certain challenges to overcome to further enhance the applicability of SP ICP-MS. In particular, matrix effects still jeopardize the accuracy of the results that are obtained when the analysis of real, complex samples is aimed at [13,14,15,16,17].

The presence of the matrix can give rise to both spectral overlaps and non-spectral interferences, and the strategies for circumventing their influence on the results are well described in the literature when using ICP-MS for elemental analysis in its “classic” operation mode: the continuous aspiration of a homogeneous solution, giving rise to *quasi*- stable signals [18]. However, the impact of such interferences on the registered signal is different for SP ICP-MS, where the instrument is operated in time-resolved analysis (TRA) mode. Therefore, additional efforts to implement adequate correction strategies are needed [19].

In this regard, the occurrence of spectral overlaps does not show the same effect in both operation modes. In SP ICP-MS, it may be possible to temporally differentiate the signals originating from the NP from those stemming from the overlapping species (unless the parent compound giving rise to such species is found within the own NP). Still, this issue typically leads to a more intense background and, consequently, to worse size limits of detection (LOD_size_). Nevertheless, such spectral interferences can be surmounted in the exact same way as the conventional ICP-MS operation mode: using high-resolution mass analyzers and/or reaction/collision cells [20,21,22].

On the other hand, non-spectral interferences may affect the sensitivity of the analytical signal, leading to either the over- or underestimation of the particle size. Additionally, the transport efficiency (TE) to the instrument (i.e., the ratio between the detected and the aspirated mass of the analyte) can also be affected, as the presence of the matrix may modify the number of registered events and, thus, bias the particle number concentration (PNC) [23]. Correction strategies, in this case, are not as straightforward as for spectral interferences, so different methodologies based on those conventionally used for ICP-MS have been proposed in the literature to cope with this issue. These strategies include matrix-matched calibration [10,24], microdroplet calibration [16,25,26], sample pre-treatments (e.g., matrix separation) [27], standard addition [19], isotope dilution [28,29], and the use of internal standards (IS) [30].

Of such strategies, IS is perhaps the simplest and more universal approach, and it is routinely applied in the conventional ICP-MS operation mode. Unfortunately, most ICP-MS instruments (e.g., quadrupole, q) work in a sequential mode and cannot simultaneously detect both the analyte(s) and the IS signals, as would be ideal for the best performance of any IS. Nevertheless, operating in a fast sequential fashion (e.g., changing the *m*/*z* every few tens of ms) is enough to benefit from the use of an IS when the signal obtained is fairly stable and lasts several seconds or even minutes. It is then assumed that no significant changes are produced in the signal when the spectrometer changes to a different *m*/*z*. Moreover, the existence of a short settling time in which the detector is not acquiring the signal after the *m*/*z* change, typically in the range of 200 µs in modern ICP-MS instruments, is not so significant in this case in comparison with all the time where the detector is actually recording the analytical signal. 

This all changes when SP ICP-MS in TRA mode is deployed. In such a case, there is not a continuous signal but a series of extremely short peaks (300–500 µs) that correspond to the detection of each single NP ion cloud. Most SP ICP-MS applications deploying state-of-the-art q-ICP-MS instruments use very short dwell times (50–100 µs), and the detector is always set at the *m*/*z* of the analyte (no settling time). This enables us to record these transient peaks with full integrity, with minimal contribution from the background (thus leading to better LOD_size_ values), no occurrence of split events (which means that one NP is detected and counted as two smaller NPs), and practically no influence from double events (two NPs counted as one bigger NP).

Using this approach, the application of IS for SP ICP-MS is not straightforward, unless a type of ICP-MS with truly simultaneous multi-isotopic monitoring potential is used (e.g., equipped with a time-of-flight (TOF) analyzer). Nevertheless, given that the use of an IS can correct not only for matrix effects but also for random errors, such as torch instabilities or instrumental drifts [18], adapting this strategy for SP ICP-MS as well can be very beneficial.

Huang et al. [30] evaluated the use of ^103^Rh as IS when characterizing CeO_2_ NPs via SP q-ICP-MS. However, they had to change the detected *m*/*z* between ^103^Rh^+^ and ^140^Ce^+^ in every measurement run (thus necessitating a settling time) and made use of long dwell times for each *m*/*z* (10 ms), which, as discussed before, do not produce the best performance for NP characterization via SP ICP-MS.

On the other hand, recent work from the group of D. Clases has demonstrated some potential benefits when operating a q-ICP-MS in bandpass mode [31,32]. This strategy maximizes ion transmission in the quadrupole (i.e., the number of ions that trespass the mass analyzer) at the expense of selectivity (i.e., the range of mass-to-charge ratios that are simultaneously detected).

Therefore, *a priori*, by means of this strategy, not only can the sensitivity be enhanced, but other species may also be detected during the same measurement run while using very short dwell time values and without modifying the *m*/*z* of the detector (no settling time needed), thus opening new possibilities for the use of an IS in q-ICP-MS, which have not been reported to date.

In this work, a novel approach for the application of IS correction in SP ICP-MS is proposed for AuNPs characterization, using the signal from PtNPs as IS. The characterization of AuNPs in complex matrices was chosen as a model to prove the performance of this approach because these are one of the most widely used metallic NPs. For this purpose, the results achieved in water media were compared to those reached in two complex media of interest: 5.0 g L^−1^ NaCl in water [33] and 2.5% (*v*/*v*) tetramethylammonium hydroxide in water [34,35,36].

## 2. Materials and Methods

### 2.1. Reagents, Standards, and Samples

Reagents of analytical purity grade were used for all experiments. Deionized water (18 MΩ cm) obtained from a Milli-Q water system (Millipore, Molsheim, France) was used throughout the work. Additionally, every sample and standard was prepared in a 1 mM trisodium citrate dihydrate (Merck, Darmstadt, Germany) aqueous solution.

For calibrating, a 1000 mg L^−1^ Au stock standard solution (Merck) was conveniently diluted, adding together, as IS, approximately 60,000 NPs mL^−1^ (calculated from the size and concentration reported by the manufacturer). NanoXact^TM^ PtNPs of 70 nm nominal size were purchased from NanoComposix Europe (Prague, Czech Republic). For TE determination, the particle size method was applied with a 50 nm AuNP standard (NanoComposix Europe) [37,38].

The performance of the method was tested for 100 nm Ultra Uniform^TM^ AuNPs (NanoComposix Europe) diluted in three different matrices: pure water, a 5.0 g L^−1^ NaCl aqueous solution (Merck), and a 2.5% (*v*/*v*) TMAH/0.1% Triton X-100 aqueous solution, with the latter prepared from GPR RECTAPUR TMAH 25% in aqueous solution (VWR chemicals BDH^®^, Fontenay-sous-Bois, France) and Triton X-100 for gas chromatography (Merck). 

### 2.2. Instrumentation

Measurements were carried out with a NexION 300X (PerkinElmer, Waltham, MA, USA) q-ICP-MS equipped with the Syngystix Nano Application module for SP ICP-MS analysis. Daily performance checks were done to adjust the sampling depth, torch position, QID, and nebulizer gas settings, so that the sensitivity was maximized while keeping the Ce^++^(70)/Ce^+^(140) and CeO^+^(156)/Ce^+^(140) ratios equal or under 0.03 and 0.025, respectively. Moreover, due to the use of the dual detection mode, daily cross-calibration of the counting to analog conversion factor is mandatory, especially given the fact that we pursued the simultaneous detection of two well-resolved nanoparticle populations that may be detected by different modes. Further instrumental details and operating conditions are summarized in Table 1.

### 2.3. Measurement Protocol

#### 2.3.1. Sample Preparation and Introduction to the ICP-MS

All NP suspensions were diluted until a total PNC (including both Au and Pt NPs) of about 60,000 NPs mL^−1^ was achieved so that the probability of double events was always below 0.01%. Additionally, every suspension and solution was prepared in 1 mM trisodium citrate to prevent the aggregation of NPs. Finally, for proving the performance of the IS correction, a 100 nm AuNP standard was suspended in three different matrices (pure water, a 5.0 g L^−1^ NaCl solution, and a 2.5% (*v*/*v*) TMAH/0.1% Triton X-100 solution) and used as sample.

Samples and ionic standards were introduced into the ICP-MS instrument through a sampling tube of 0.38 mm internal diameter (ID), using a peristaltic pump adjusted so that a constant uptake rate (Q) was maintained around 0.32 mL min^−1^, according to gravimetric measurements carried out prior to every session of analysis. Such measurements were repeated at the end of each session to assess the stability of Q, but no significant difference was observed.

#### 2.3.2. Analysis and Calibration

**Figure 1:**
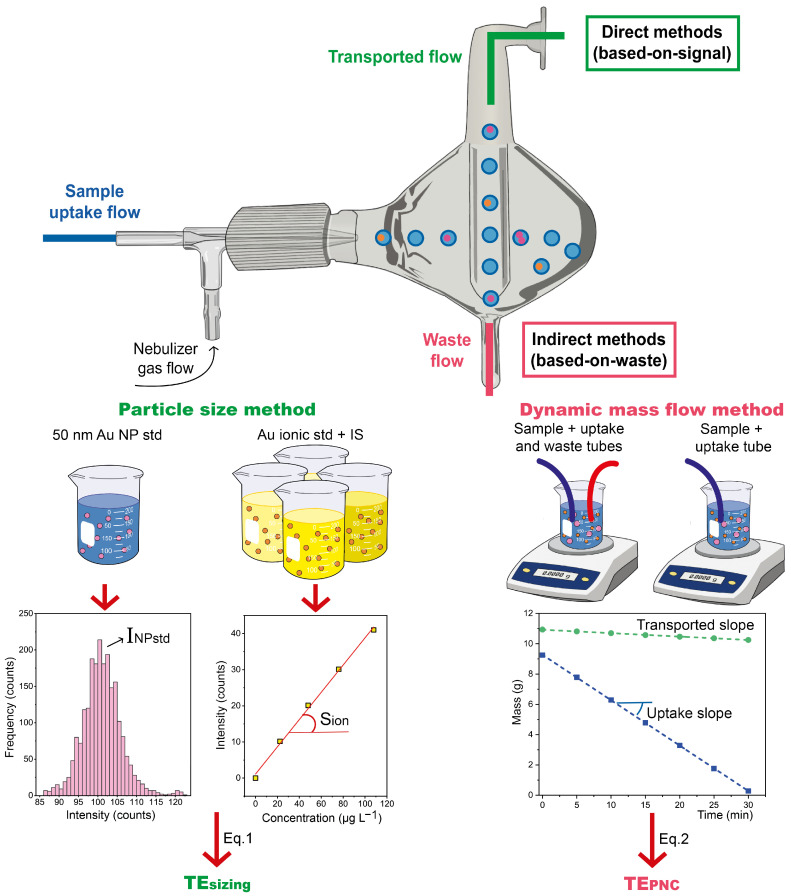
Scheme of the measurement protocol followed to determine TE_sizing_ via the particle size method (**left**), and TE_PNC_ via the dynamic mass flow method (**right**).

**Figure 2:**
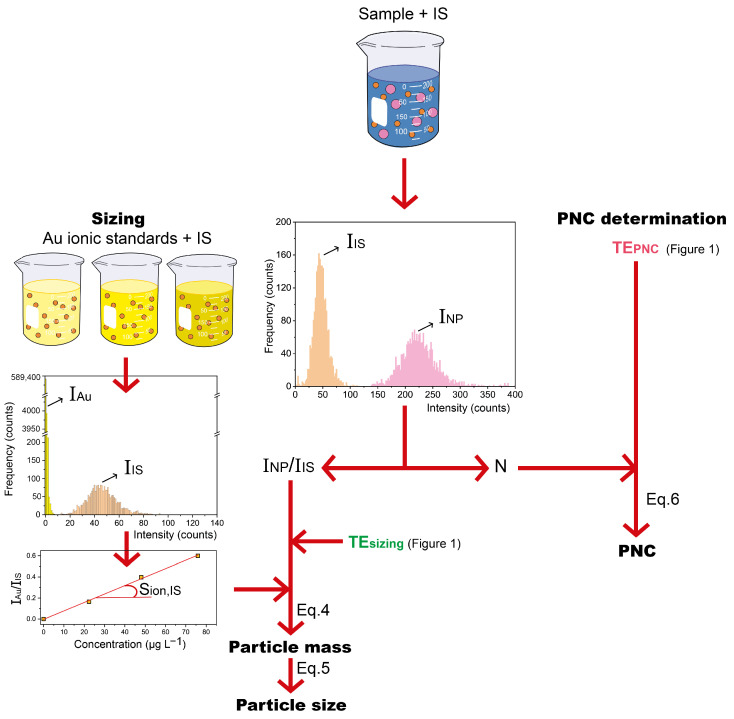
Measurement protocol followed to determine the particle size and PNC of AuNP suspensions spiked with PtNPs as IS. The calculations are carried out according to Section 2.3.2.

Measurements were carried out in quintuplicate for ionic standards and in triplicate for sample dispersions (given the necessity of longer acquisition times in this case, as will be discussed later on). The measurement protocol consisted of two steps: first, the determination of the TE, and second, the analysis of the sample. These steps are described below and schematized in Figure 1 and Figure 2, respectively.

For the determination of the TE, as is further discussed in Section 3.2, two different methods were used: the particle size method and the dynamic mass flow method [37,38,39]. The former was employed to obtain the transport efficiency for sizing the NPs (*TE_sizing_*), whereas the latter method was performed to determine the transport efficiency to calculate the PNC (*TE_PNC_*).

Regarding the particle size method, a 0.23 ng mL^−1^ suspension of AuNPs of reference size of 50.3 nm was introduced into the ICP-MS instrument. The monitored signal was processed to determine the average NP intensity in counts ($I_{NPstd}$), as described in Section 2.3.3. Next, a calibration curve prepared with ionic Au standards of concentration between 0 and 70 ng mL^−1^ was measured, and the slope of the curve was calculated in counts mL ng^−1^ ($S_{ion}$). Finally, the *TE_sizing_* was calculated according to Equation (1), where *Q* is the uptake flow in mL min^−1^, 60 is the minutes to seconds conversion factor, $t_{dwell}$ is the dwell time in s, and $m_{NPstd}$ is the reference average mass per NP of the standard in ng.

(1) $$TE_{sizing}=\frac{\frac{S_{ion}}{Q\;\frac{t_{dwell}}{60}}}{\frac{I_{NPstd\;}}{m_{NPstd}}}$$

For the dynamic mass flow method, *TE_PNC_* was calculated in accordance with Equation (2). The *Uptake slope* was obtained by tracking and representing the evolution of the sample mass over time while aspirating each sample. The *Transported slope* was determined analogously but introducing in the sample vial both the sampling and the waste tube.

(2) $$TE_{PNC}=\frac{Transported\;slope}{Uptake\;slope}\;\;$$

After determining the *TE_sizing_*, particle mass calculations for the samples would be straightforward via Equation (3) since the only unknown value is the intensity of the *NP* sample in counts ($I_{NP}$). Notwithstanding, results are expected to be biased due to matrix effects, so samples and standards were spiked with a concentrated *IS* suspension until the target total *PNC* (i.e., the sum of the *PNC* for Au and PtNPs) of 60,000 NPs mL^−1^ was reached (note that in order to avoid the occurrence of double *NP* events, lower spike concentrations are necessary for *NP* samples than when in the absence of NPs, as is the case for ionic standards). To this end, Equation (3) can be derived into Equation (4), where $I_{NP}/I_{IS}$ is the quotient between the intensity of the *NP* sample and that of the *IS*, and $S_{ion,\;IS}$ is the slope of the calibration curve obtained when plotting the quotient between the average ionic intensity and $I_{IS}$ against the ionic concentration.

(3) $$m_{NP}=\frac{I_{NP}\;t_{dwell}\;Q\;TE_{sizing}}{S_{ion}}\;$$

(4) $$m_{NP,IS}=\frac{\frac{I_{NP}}{I_{IS}}\;t_{dwell}\;Q\;TE_{sizing}}{S_{ion,\;IS}}$$

For sizing purposes, particle mass results can be converted to diameters via Equation (5), introducing the estimated particle density in g cm^−3^ and the corresponding particle mass (*m*) in ng (calculated via Equation (3) or (4)).

(5) $$d_{NP}=\sqrt[{3}]{\frac{6}{\pi }\frac{m}{\rho_{NP}}}10^{4}$$

Finally, the *PNC* in the aspirated sample can be easily determined by introducing the number of registered events per minute (*N*) in Equation (6), where *Q* is again the uptake flow in mL min^−1^.

(6) $$PNC=\frac{N}{TE_{PNC}\;Q}$$

#### 2.3.3. Data Processing

For data processing, a script developed in-house with GNU Octave was employed, as described elsewhere [19]. In short, such program identifies (according to a 5σ criterion) and integrates (after subtracting the average ionic contribution of the medium) the peaks of raw data taken from the instrumental software so that they can be further processed and represented with the OriginPro software (version 2021b, 9.85). NP histograms were fitted to Gaussian functions, and their central values were used as analytical signal ($I_{NP}$). For the calibration, the average background intensity subtracted from the NP events was taken as ionic signal ($I_{ion}$).

## 3. Results and Discussion

### 3.1. Optimization of the Quadrupole Resolution

As discussed in the introduction, depending on the voltage settings applied to the quadrupole, it is possible to adjust the ion transmission and the mass resolution of the spectrometer. In conventional work with ICP-MS, selectivity is generally favored over sensitivity to minimize the risk of spectral overlap. However, operating in TRA represents different challenges. In this sense, selectivity requirements are lower, as the high temporal resolution achieved is advantageous to differentiate the signal stemming from the analyte in the NPs while enhancing the sensitivity enables the characterization of smaller NPs. Moreover, the risk of spectral overlap at some high *m*/*z* is often limited, and, as will be evaluated in this work, there is the possibility to transform such potential overlap into positive information, i.e., an IS signal.

In particular, we propose in our investigation the addition of PtNPs to serve as IS for the characterization of AuNPs. By opening the bandpass in the quadrupole, it should be possible to monitor both Au and Pt in the same run without modifying the *m*/*z* settings of the quadrupole. Please note that the detection of both types of NPs would not be truly simultaneous, as the idea is to differentiate them by the magnitude of the signals stemming from each type of NP. As explained in the experimental section, the suspensions must be highly diluted to minimize the risk of both types of NPs arriving exactly at the same time, as it would not be possible to separate their signals.

This is also the reason to choose NPs as IS and not an ionic species. The ionic species would produce a constant BG that would deteriorate the LOD for size characterization, which will not occur when adding NPs. Moreover, it will not be possible to differentiate the signals originating from Au or Pt ions when operating in bandpass mode, thus impeding the calibration of the sensitivity for sizing the target NPs. On the other hand, when using NPs as IS, since very short discrete events will be recorded, it may be possible to obtain separate distributions for the IS (Pt in this case) and the target NP (Au in this case).

**Figure 3:**
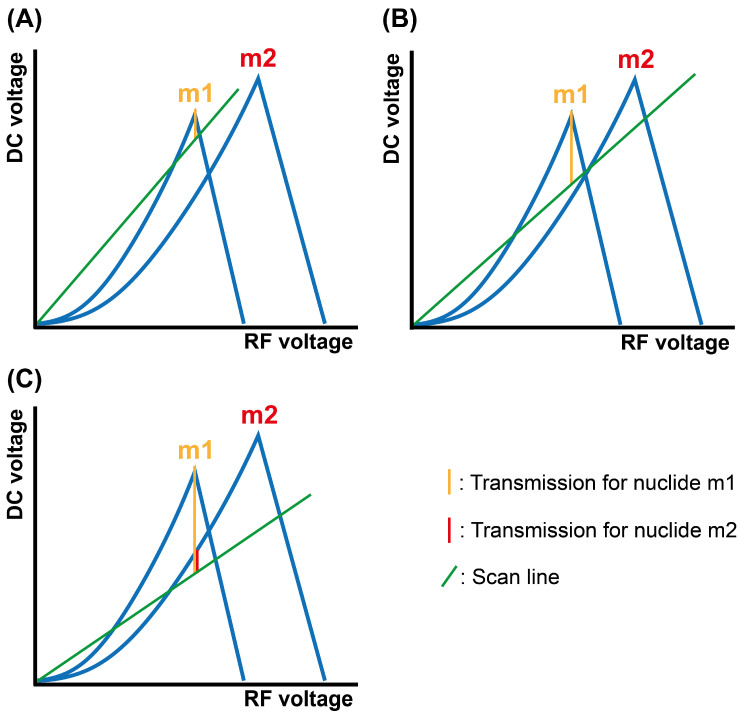
Schematic representation of the stability diagram of two nuclides of different *m*/*z* ratio, m1 and m2, in a quadrupole mass spectrometer. The figure shows the differences between their transmission (factor that affects the sensitivity) for high (**A**), low (**B**), and very low (**C**) DAC values.

To achieve this multiple detection, the parameter to be tuned for the instrument used in this work is the digital-to-analog converter (DAC) value, which modifies the DC/RF ratio applied to the quadrupole, which in turn defines ion transmission and quadrupole resolution. Under normal circumstances, the DAC value is set to about 2060 to achieve a resolution of approximately 1 u, making sure that only the *m*/*z* of interest is being monitored. However, this parameter can be manually modified in the mass calibration window of the instrumental software to measure a different *m*/*z* range. The performance of the DAC value is represented in Figure 3, where the transmission achieved for two nuclides of different (but close) *m*/*z* ratios (m1 and m2) is compared for different quadrupole scan lines. For higher DAC values (Figure 3A), the nuclide focused (m1) is selectively monitored at the expense of sensitivity. Upon reducing the DAC value, the ion transmission (and, thus, the sensitivity) is improved for this nuclide (m1) (Figure 3B) until, under a certain value, the scan line penetrates the stability diagram of the second nuclide (m2), which is, in turn, simultaneously detected with m1 (Figure 3C).

**Figure 4:**
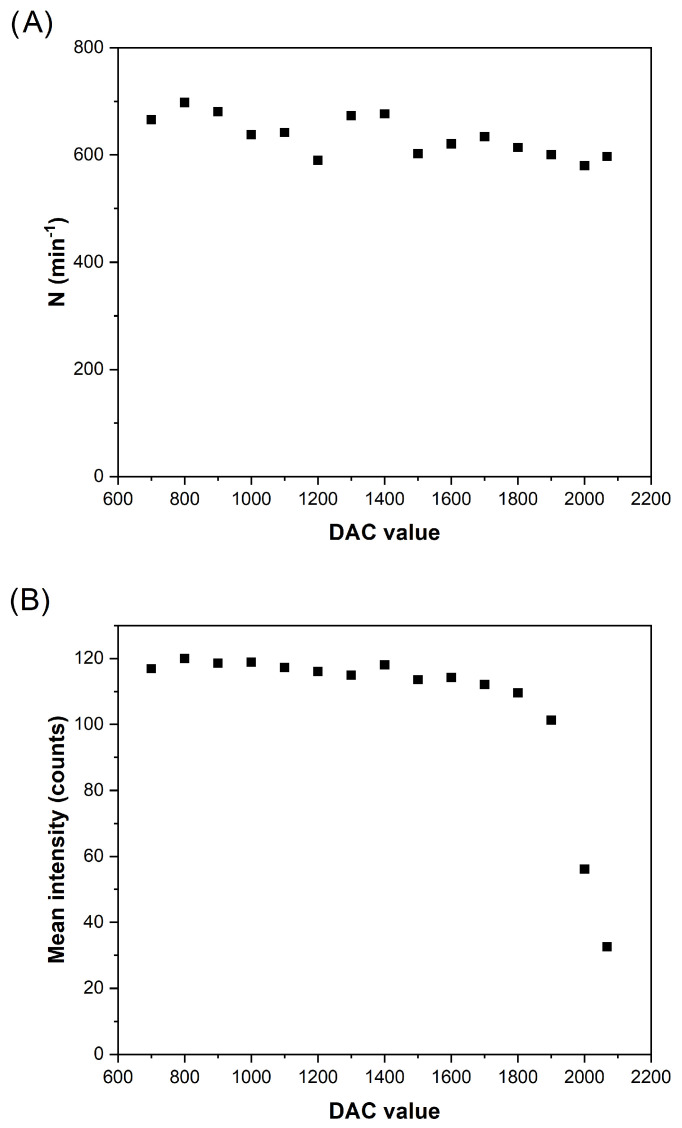
Evolution of the number of events detected per minute (**A**), and mean intensity (**B**) obtained for a distribution of AuNPs (100 nm, 60,000 particles per mL) as a function of the DAC value used for SP ICP-MS measurements.

To optimize this parameter, an aqueous suspension of 100 nm AuNPs was monitored at different DAC values, setting the *m*/*z* at 197 in the instrument software. Next, the NP distributions obtained (in counts) were represented and were fitted to Gaussian functions. To evaluate the results, the number of events detected per minute (N), as well as the central value of the fitted Gaussians were calculated. The evolution of these variables is shown in Figure 4. As expected, no clear tendency is appreciated for N, as we are working well above the LOD in all cases. In any case, the intensity recorded per event is enhanced when decreasing the DAC parameter, until a plateau is achieved for values below 1800. Below this value, no further sensitivity improvement is reached, meaning that the transmission of the ^197^Au^+^ ion is maximum.

**Figure 5:**
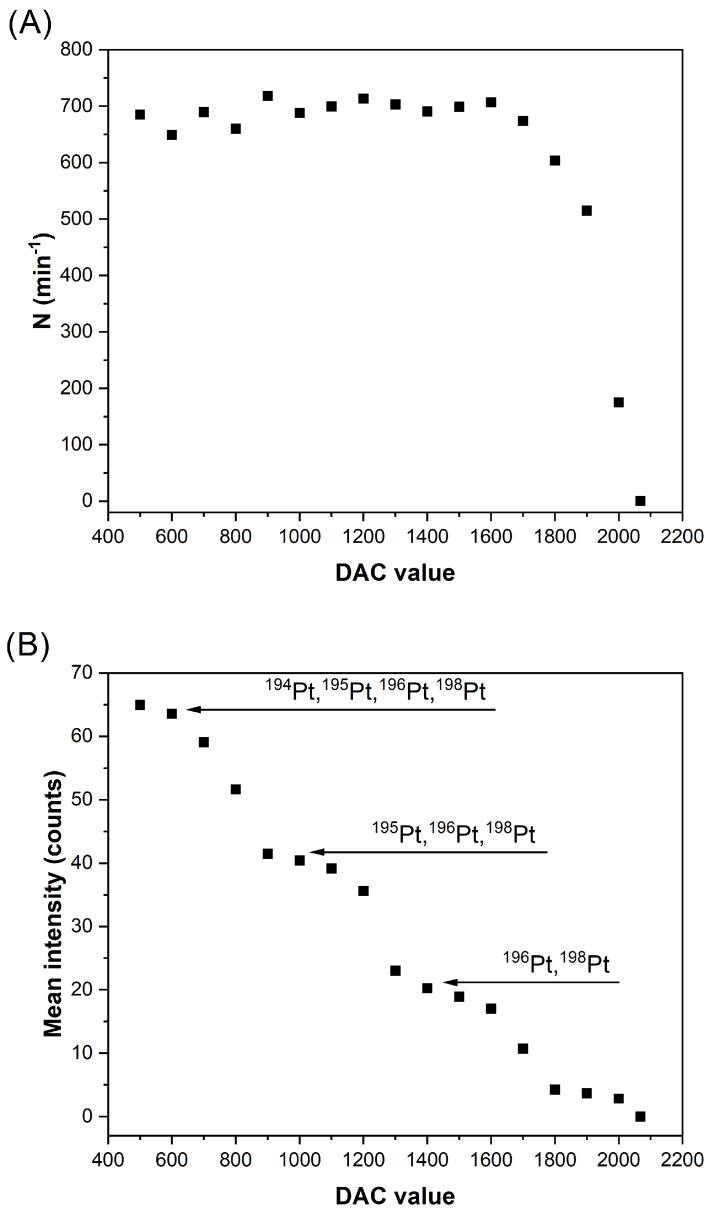
Evolution of the number of events per minute (**A**) and mean intensity (**B**) for PtNPs (70 nm, 60,000 particles per mL) as a function of the DAC value. The lower mean intensity step probably corresponds to the detection of ^198^Pt; however, this fact could not be experimentally verified since the NP distribution was not sufficiently separated from the background. Acquisition performed by setting the *m*/*z* at 197.

The same experiment was also conducted for a suspension of 70 nm PtNPs, providing the results shown in Figure 5. In this case, these NPs are detected when using low DAC values, as the bandpass is open, even if we are setting the quadrupole at a *m*/*z* value of 197. N is unaffected by the DAC until, for values above 1700, it acutely decays. This effect is due to the fact that for DAC values above 1700, the *m*/*z* = 197 is more selectively focused, and Pt ions are not effectively transmitted through the quadrupole. Regarding the sensitivity, a laddered evolution is registered, observing four intensity plateaus that can be well correlated to the complete transmission through the quadrupole of the four main Pt isotopes, as indicated in Figure 5B. This hypothesis was confirmed by comparing the experimental intensity ratios to the natural abundance ratios, as shown in Table 2.

For the further development of the method, the DAC value was set to 1500 for two reasons. First, it corresponds to the center of one of the steps of the laddered evolution curve, so it can be expected that results will be less affected by potential instrumental drifts of the DAC parameter. Second, it provides one of the lowest intensity values for the PtNPs (but still above the LOD), so the IS distributions will be more likely to be fully resolved from those obtained for the sample dispersions (100 nm AuNPs).

This optimization procedure should be done for every new situation where the size of the target NPs and that of the NPs used as IS change. It should be remarked that one of the strengths of the method is that, as shown in Figure 5, very different sensitivities can be achieved for the PtNPs used as IS by simply tunning the DAC parameter. Therefore, depending on each application investigated (e.g., for different Au and Pt particle sizes), a different DAC value will be optimal to ensure fully resolved distributions. In this vein, it is also important to remind that a preliminary measurement of the sample in the absence of IS is always necessary in order to identify which distribution corresponds to the analyte and which one to the IS.

### 3.2. Determination of Transport Efficiency

For the determination of the transport efficiency (TE), several protocols have been proposed in the literature, which are classified either as direct or indirect methods [39]. The former group consists of approaches in which the TE is calculated from the signal registered. On the other hand, indirect methods are based on determining both the transported and waste flows, while the TE is calculated as their quotient. All these approaches have their pros and cons, and the suitability of each method depends on both the particular analytical problem considered and the accessibility to certified NP standards [38]. In this sense, direct methods are most often deployed, but they require NP standards certified in either the PNC for the particle frequency approach or in size for the particle size method.

The particle size method tends to provide the best results when calculating the size of the NP suspensions [13,37], as it is really not a pure TE method, and it factors in other aspects that may affect the sensitivity as well. Thus, it was adopted in this work for sizing the NPs. As described in Section 2.3.2, (see Equations (3)–(6)), matrix effects were dealt with by introducing the PtNPs as IS. It is important to stress that for these calculations, it is not necessary to know either the exact size or the exact PNC of these PtNPs used as IS, as only the central value of the PtNP distribution obtained experimentally (in counts) is used for the calculations. Thus, the only requirement in practice is that the PtNP size, whatever that value is, does not vary during the measurements. It is true, though, that the achievement of such requirement can be eased by the use of monodisperse NP standards, as they lead to narrower signal distributions that are not only easier to separate from those of the analyte, but also exhibit more consistent central tendencies. 

The determination of the PNC is often more challenging for SP ICP-MS [40]. In principle, the particle frequency method is conceptually simple to use, and it is possible to estimate the TE by this method even with NPs of a different analyte [19]. This would allow us, for instance, to use the NPs deployed as IS as a standard for the determination of the TE. For this purpose, however, a reliable PNC value should be known in advance for these NPs, and this is not very often possible. Thus, to estimate the TE for determination of the PNC (Equation (6)), a different, indirect approach was selected: the dynamic mass flow method first proposed by Cuello et al. [41], which requires no knowledge of the PNC of any suspension of NPs.

As to the results, the particle size method was performed with the standard of monodisperse 50 nm AuNPs in water, obtaining a TE_sizing_ of 7.1%. The differences induced by the matrix were corrected for by the IS, as supported by the good results obtained for the sizing of AuNPs, as will be shown in Section 3.3. On the other hand, for the indirect method, each sample was injected, and the transport and uptake rates were determined by weighing (see Figure 1), leading to different TE_PNC_ for the aqueous, NaCl, and TMAH/Triton matrices, as expected: 7.2 ± 0.1%, 6.6 ± 0.1%, and 7.5 ± 0.2%, respectively (uncertainty budgets calculated as the combined uncertainties of the uptake and transported flow slopes). It is worth commenting at this point that using these values in Equation (3) for the sizing of AuNPs will lead to biased results, as will be discussed in Section 3.3. This supports the idea that indirect methods to determine the TE are particularly well suited for obtaining accurate PNC values [39].

### 3.3. Characterization of AuNPs in Different Matrices

One of the main interests in using an IS would be the ability to compensate for matrix effects. Thus, two complex but typical media were studied. First, an aqueous solution with 5.0 g L^−1^ NaCl was tested. This matches the saline content expected in biological samples [33] while introducing a high amount of an easily ionized element that could lead to potential matrix effects in the plasma. Additionally, an aqueous solution containing 2.5% (*v*/*v*) tetramethylammonium hydroxide (TMAH)/0.1% Triton X-100 was also tested, which is one of the most popular media for the extraction of NPs from biological tissues [34,35,36] and has already been shown to produce matrix effects in SP ICP-MS [19,41].

To prepare the samples, 100 nm AuNPs and 70 nm PtNPs standards were suspended in the three matrices, maintaining a PNC around 30,000 NPs mL^−1^ for each of them so that the total PNC is the optimum value to ensure that the probability of double events is lower than 1%. To achieve the same number of NP events (of each type) counted per measurement, the acquisition time was doubled to 200 s.

To prove the performance of the proposed IS correction, the sizing results achieved for the 100 nm AuNPs via direct and IS calibration were compared. It is noteworthy that both calibration curves were constructed with standards prepared by the dilution of ionic Au standards in the presence of PtNPs. However, for the direct approach, only the signal of the ionic background was considered, whereas for the IS calibration, such signals were divided by the NP intensity ($I_{IS}$). Diameters characterized by both approaches for each sample are summarized in Table 3, where they are compared to the reference size (102.2 nm) via Student’s *t*-test. The results indicate that the external calibration (no IS correction) only provides sizes not significantly different from the certificate for the water media, where matrix effects are absent. On the other hand, the IS correction leads to unbiased diameters regardless of the matrix content, which evidences the possibilities of this approach.

It is necessary to indicate that the reason for performing a one-sample contrast analysis in this case is that the reference uncertainty gathered in the certificate of analysis for the AuNPs characterized represents the dispersity of the NP distributions, while the uncertainty of the results obtained via SP ICP-MS represents the dispersion of the mean size determined, and thus, they cannot be statistically compared. Thus, the SP ICP-MS values are tested against the reference value only (102.2 nm).

**Figure 6:**
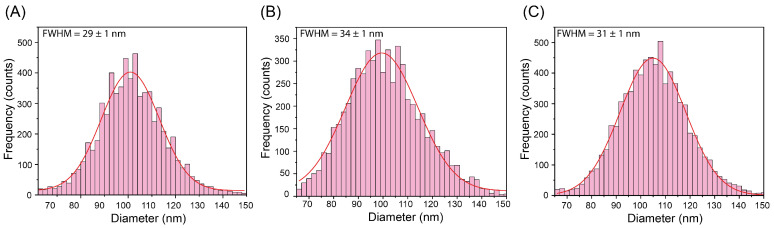
Size distributions and fitted Gaussian functions achieved via IS correction for 100 nm AuNPs in pure water (**A**), aqueous NaCl (**B**) and aqueous TMAH/Triton (**C**) matrices. The FWHM are indicated to compare the dispersity of the fitted Gaussians. Uncertainties expressed as the standard deviation of the Gaussian fitting.

The size distributions were very similar for each matrix, which portrays the good performance of the IS correction, as shown in Figure 6. These distributions suggest a wider size dispersity than the reference value (102.2 ± 4.2 nm), which, assuming a Gaussian distribution, would correspond to a full width at half maximum (FWHM) of 9.9 nm. This widening has already been reported in previous SP ICP-MS works and may be due to sample stability issues and/or the technique itself [19].

Regarding PNC determination, the results achieved for each sample with their corresponding TE_PNC_ were compared to the reference value provided by the manufacturer (4.9 × 10^9^ mL^−1^), as shown in Table 4. Additionally, results that would have been achieved using TE_sizing_ instead were also collected to ratify the necessity of two separate TE determinations. As for the results, unbiased concentrations were achieved for every matrix when using the corresponding TE_PNC_, whereas TE_sizing_ only provided unbiased results in the absence of matrix effects, as expected. Finally, it should be acknowledged that the AuNP standard used as a sample was not certified in PNC, so the real concentration may be a bit different from that calculated in the certificate. Similar results were achieved when using TE_PNC_, regardless of the matrix, suggesting that it is the most accurate methodology.

Finally, one could suggest the use of the corresponding TE_PNC_ of each sample for the direct determination of the particle size, so neither the IS nor the calculation of two different TE is required. However, and as discussed in Section 3.2, matrix effects do not necessarily impact the number of detected events and the sensitivity to the same extent, and, moreover, the dynamic mass flow method is particularly developed for PNC measurements [39]. In this regard, diameters of 93.6 ± 1.8 nm and 121.4 ± 1.6 nm would be achieved in this way for the NaCl and TMAH/Triton matrices, respectively. These results are, evidently, significantly different from the reference value (P_0_ of 0.015 for NaCl and 0.002 for TMAH/Triton), which confirms the necessity of the two different methods for the TE determinations.

## 4. Conclusions

In this paper, an IS correction was developed for the characterization of AuNPs by SP ICP-MS. For this purpose, the use of the quadrupole bandpass mode enabled the detection of both Au and PtNPs during each measurement run, so one of them (Pt in this case) can serve as IS for the other. Additionally, via this operation mode, it is possible to tune the sensitivity of PtNPs to achieve signal distributions sufficiently resolved from those of AuNPs and, also, increase the sensitivity of the analyte. The performance of the developed methodology was proofed for three different matrices: pure water, a 5.0 g L^−1^ NaCl water solution, and a 2.5% (*v*/*v*) TMAH/0.1% Triton X-100 water solution, exhibiting good performance for compensating for matrix effects appearing for the NaCl and TMAH/Triton solutions.

In comparison to other correction strategies, the proposed methodology offers several advantages, in a similar way as conventional ICP-MS. First, unlike approaches based on matrix-matched calibration, no previous knowledge of the matrix composition is required. Second, it is faster and more straightforward than methods that include pre-treatment steps to remove non-spectral interferences. Finally, it is less tedious than standard addition approaches and more universal than isotope dilution. Moreover, even if the IS is introduced in both samples and calibration standards, it is always possible to register the analyte signal only by simply modifying the bandpass parameters of the quadrupole, and thus other approaches may always be used later on.

As for the NP characterization, two different methods for TE determination were employed: the particle size method for sizing (TE_sizing_) and the dynamic mass flow method for the PNC determination (TE_PNC_). Once the TEs were obtained, the size and PNC results achieved by external calibration and by the proposed method were compared, concluding that only the IS approach led to unbiased result regardless of the matrix composition. It is also important to stress that for the approach proposed, it is not necessary to know beforehand either the size or the PNC of the suspension of NPs used as IS.

Finally, it is noteworthy that the same approach could be applied to characterize PtNPs using AuNPs as IS, but it might also be easily adapted for different couples of NPs, such as Ag and PdNPs. However, the method may not be so straightforward for species of lower *m*/*z*, as they are more affected by spectral interferences. For such cases, the analysis could be eased using an ICP-MS/MS instrument [42,43]. Moreover, the bandpass mode would enable the simultaneous (in the same measurement run) characterization of both Pt and AuNPs when in the absence of matrix effects (no IS required). Other applications may also be possible using the proposed approach.

## Figures and Tables

**Table 1:** ICP-MS operation conditions.

Measurement Conditions	
RF power, W	1450
Nebulizer gas flow, L min^−1^	0.65
Plasma gas flow, L min^−1^	15
Auxiliary gas flow, L min^−1^	1.2
*m*/*z*	197
DAC parameter	1500
Detection mode	Dual (counting + analog)
Dwell time, µs	50
Acquisition time, s	100 for ionic standards 200 for NP suspensions
**Instrumental setup**	
Sample introduction system	Quartz cyclonic spray chamber Type A concentric nebulizer
Sampling tube internal diameter, mm	0.38
Uptake rate, mL min^−1^	0.32
**NP parameters for calculation**	
AuNP density, g cm^−3^	19.3

**Table 2:** Comparison of the theoretical (calculated from their natural abundance) and experimental ratios (obtained as the quotient between registered intensities at different *DAC* values) of the Pt isotopes involved in each step of the laddered intensity evolution shown in Figure 3B.

Isotope Ratio	Experimental Equation	Experimental Ratio	Theoretical Ratio
^194–198^Pt/^195–198^Pt	IDAC=500IDAC=900	1.53	1.50
^194–198^Pt/^196,198^Pt	IDAC=500IDAC=1400	3.13	3.06
^195–198^Pt/^196,198^Pt	IDAC=900IDAC=1400	2.04	2.04

**Table 3:** Results for the sizing of AuNPs via external calibration and with IS correction. Diameters are compared to the reference value of the standard (102.2 ± 4.2 nm) with a one-sample Student’s *t*-test, leading to the collected P_0_ values that indicate the probability of differences being due to random errors (P_0_ values below 0.05 indicate that results are significantly different). Uncertainties are expressed as standard deviations (*n* = 3).

Matrix	External Calibration		IS Correction	
	Diameter (nm)	P_0_	Diameter (nm)	P_0_
Water	103.4 ± 1.1	0.20	100.8 ± 1.1	0.14
NaCl	96.3 ± 1.9	0.03	100.4 ± 2.0	0.26
TMAH/Triton	118.7 ± 1.6	0.00	104.5 ± 1.3	0.08

**Table 4:** PNCs calculated with Eq. 6 for AuNPs via external calibration (using the same TE as for sizing, TE_sizing_) and the approach proposed in this work (using a specific TE for each sample, TE_PNC_). Results are compared to the reference value of the standard’s certificate (4.9 × 10^9^ mL^−1^) with a one-sample Student’s *t*-test, leading to the collected P_0_ values. Uncertainties are expressed as standard deviations (*n* = 3).

Matrix	TE_sizing_		TE_PNC_	
	PNC (mL^−1^)	P_0_	PNC (mL^−1^)	P_0_
Water	5.01 × 10^9^ ± 0.05 × 10^9^	0.06	4.91 × 10^9^ ± 0.05 × 10^9^	0.76
NaCl	4.70 × 10^9^ ± 0.06 × 10^9^	0.02	4.99 × 10^9^ ± 0.06 × 10^9^	0.14
TMAH/Triton	5.33 × 10^9^ ± 0.11 × 10^9^	0.02	4.98 × 10^9^ ± 0.10 × 10^9^	0.30

## Data Availability

Data will be available through Zenodo.
